# You are never too old for a congenital disease!

**DOI:** 10.3402/jchimp.v3i3-4.22091

**Published:** 2013-12-17

**Authors:** Mukul Khanna, Saro Sarkisian, Phu Tran, Ibrahim I. Ghobrial

**Affiliations:** Department of Internal Medicine, University of Pittsburgh Medical Center – McKeesport Hospital, Pittsburgh, PA, USA

**Keywords:** dysphagia, chest pain, extrinsic esophageal compression, aberrant right subclavian artery, barium esophagogram, congenital anomaly

## Abstract

Congenital diseases are sometimes overlooked by physicians because of their rarity or because of late onset of symptoms, which may delay treatment plans. This is illustrated in our patient who presented with dysphagia along with chest pain and who was found to have a congenital vascular anomaly, detected in her fifth decade of life.

A 57-year-old African American woman presented with a 1-day history of atypical substernal chest pain which was intermittent, non-pleuritic, and non-exertional. She also reported a 3-month history of dysphagia (predominantly for solids) which she described as ‘food stuck in my throat’. In addition, our patient reported a 20-pound weight loss over 3 months. She had occasional nausea and heartburn, however she denied any vomiting, odynophagia, abdominal pain, or regurgitation of food. Her medical history was significant only for gastroesophageal reflux disease. She denied any tobacco, alcohol, or drug use. She was not on any prescribed medications.

Physical examination revealed cachexia but was otherwise unremarkable. Vital signs were within normal limits. Initial evaluation showed non-specific electrocardiographic (ECG) abnormalities in the form of T-wave inversions in the lateral leads; however, she had two negative troponin assays. Her chest X-ray was unremarkable. The rest of her laboratory tests including complete blood count (CBC), basic metabolic profile (BMP), and liver function tests (LFTs) were within normal limits. The patient underwent an esophagogastroduodenoscopy (EGD), which revealed a non-bleeding gastric ulcer. Campylobacter-like Organism (CLO) test was positive for *Helicobacter pylori*, for which she received standard triple therapy. Her dysphagia was further evaluated with a barium swallow, which showed smooth extrinsic compression on the posterolateral aspect of the esophagus (Fig. 1). As the patient continued to have chest pain, she underwent a nuclear pharmacological stress test, which was high probability for ischemia. She was then taken for cardiac catheterization.

**Fig. 1:**
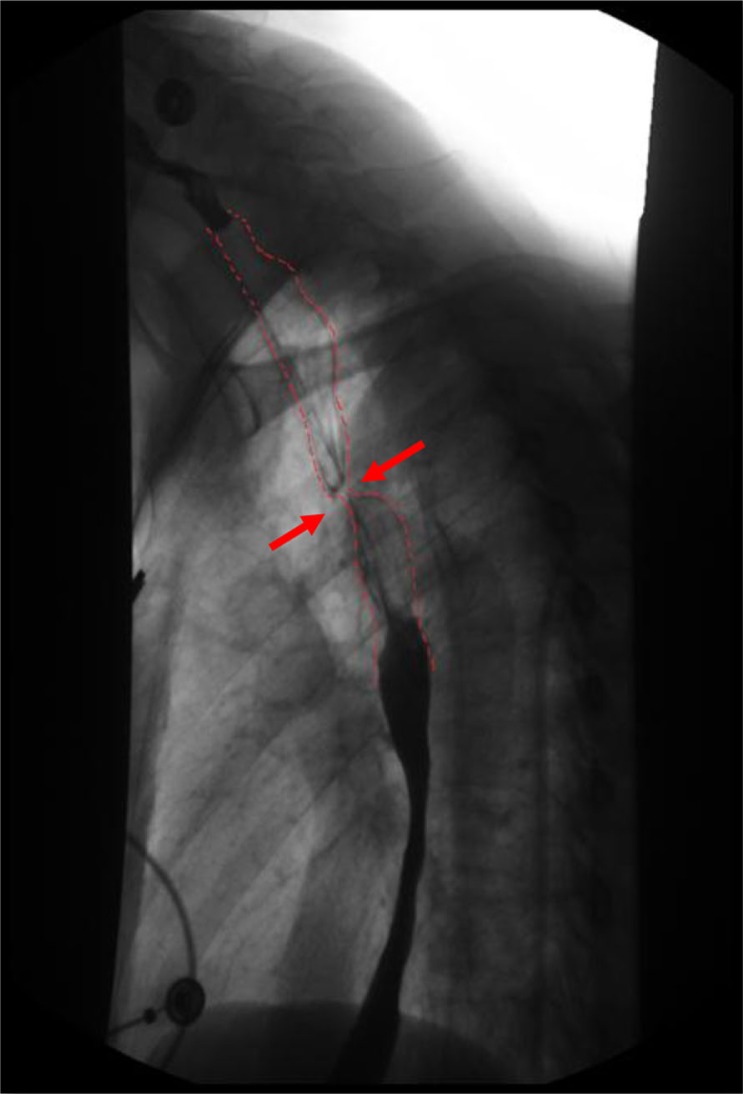
Barium esophagogram showing the compression of the esophagus (dotted red lines).

Cardiac catheterization revealed non-obstructive coronary artery disease. Due to the presence of extrinsic compression of the esophagus on the barium esophagogram, an aortogram was performed which showed an anomalous take-off of the right subclavian artery distal to the left subclavian artery (Fig. 2). This condition is known as dysphagia lusoria. In pursuing the workup of her chest pain, a computerized tomography (CT) angiogram was performed which confirmed the anomalous right subclavian artery coursing posteriorly resulting in smooth extrinsic compression of the esophagus (Fig. 3). There was no evidence of pulmonary embolism.

**Fig. 2:**
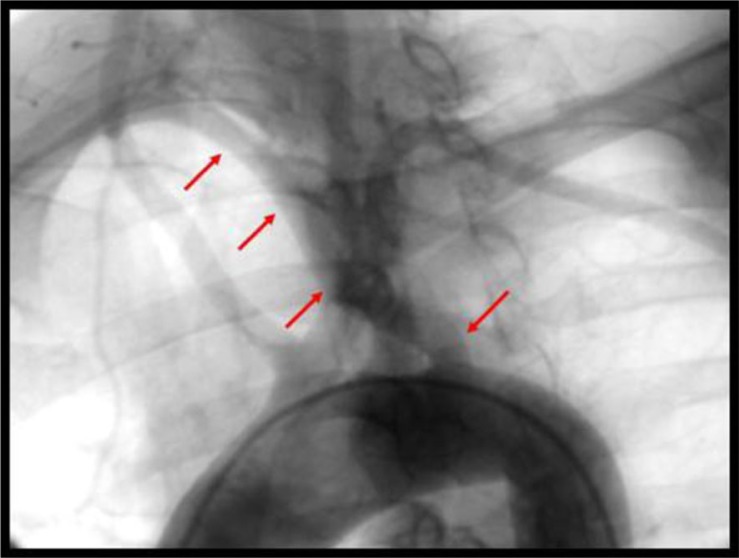
Angiogram showing the aberrant right subclavian artery originating distal to the left subclavian artery, coursing toward the right upper extremity (red arrows).

**Fig. 3:**
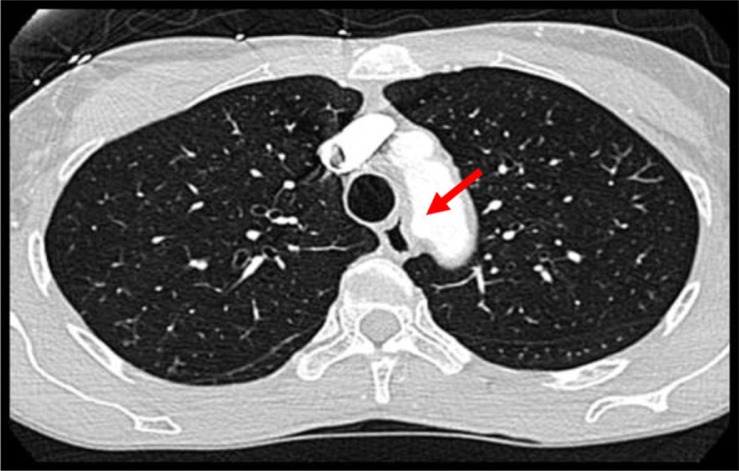
CT chest with contrast showing the origin of the aberrant right subclavian artery (red arrow) causing extrinsic compression of the esophagus.

## Discussion

**Fig. 4:**
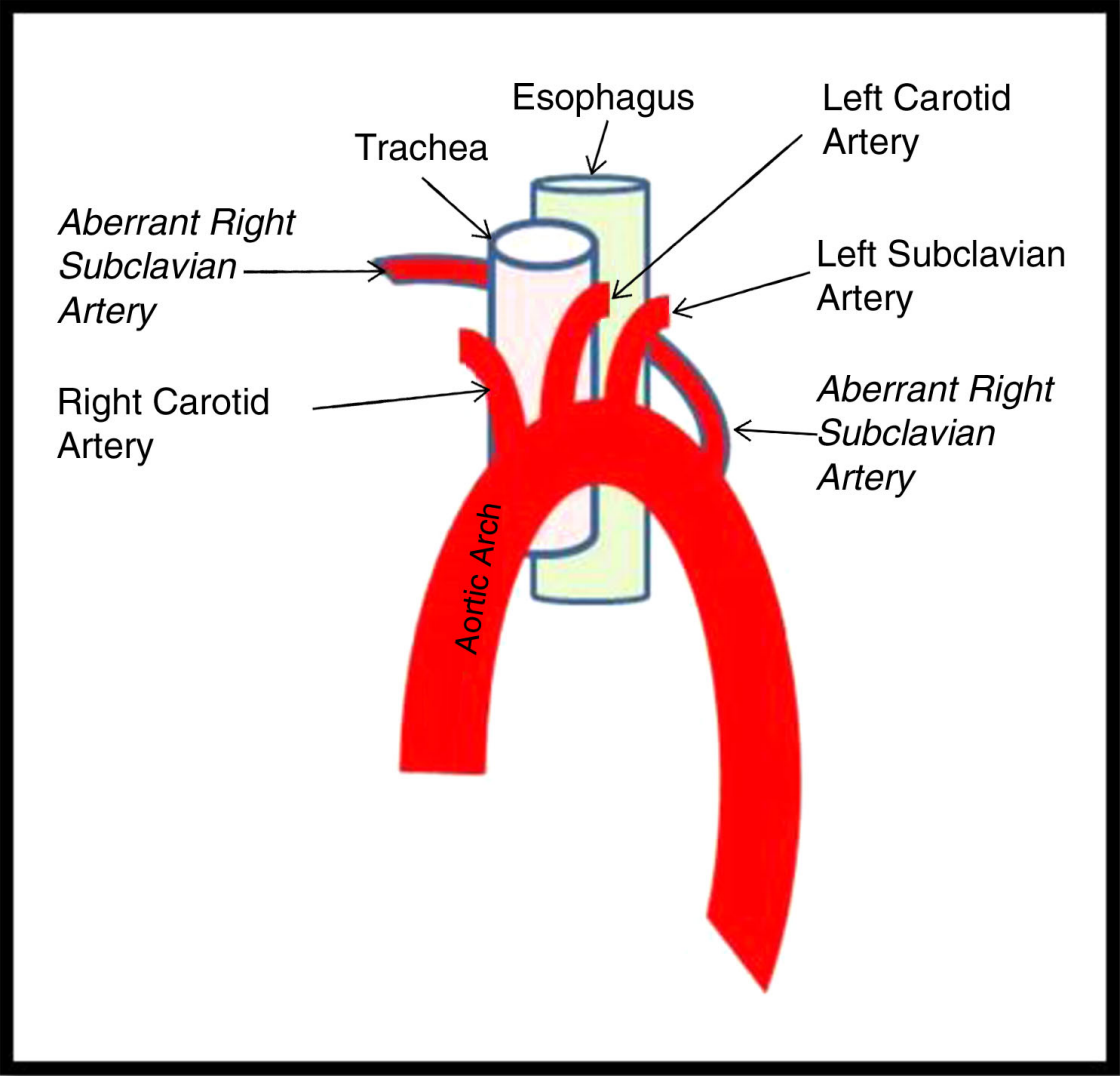
Diagrammatic representation of the anomaly.

David Bayford first described dysphagia lusoria in 1794 as Lusus Naturae or a ‘freak of nature’. It is the most common embryologic anomaly of the aortic arch, occurring in about 0.5–1.8% of the population (1). Normally, the first branch of the aorta is the brachiocephalic trunk also known as the innominate artery. It branches into the right common carotid and the right subclavian arteries. The second and the third branches are the left common carotid and the left subclavian arteries, respectively. Dysphagia lusoria is an embryological anomaly in which there is an aberrant origin of the right subclavian artery distal to the left subclavian artery (Fig. 4). The aberrant artery crosses either posterior (85%) or anterior (15%) to the esophagus causing extrinsic compression as it courses toward the right upper extremity (2, 3).

Patients with dysphagia lusoria often present with one or more of the following symptoms: dysphagia (especially to solids), chest pain, cough, hoarseness, weight loss, and anorexia. The majority of patients with dysphagia lusoria remain asymptomatic. Interestingly, patients may present with symptoms as early as in their 20s or as late as 60s (3–5). Several explanations have been suggested to explain the late onset of symptoms including arteriosclerosis/rigidity of the vessel wall, aneurysmal dilatation of the vessel, or decreased flexibility of the esophagus (6).

The management of dysphagia lusoria depends on the severity of symptoms. In mild cases without weight loss, conservative management with dietary modification should be attempted first, which includes frequent small bites, adequate mastication with sips of liquids, and use of non-solid nutritional supplements (4).

Patients who experience moderate to severe symptoms should be considered for surgical treatment. The goal of surgery is to relieve the external esophageal compression while maintaining circulation to the right upper extremity. Relief of the esophageal compression is achieved by resection of the aberrant artery. Restoration of the right upper extremity is maintained by either anastomosis of the native vessel to the right common carotid artery or by use of a synthetic graft. As our patient had severe symptoms with significant weight loss, she was referred for vascular surgical intervention.

The right supraclavicular surgical approach has been shown to be the most successful. A small transverse right supraclavicular incision is made and a mediastinoscope is used to identify the aberrant right subclavian artery. The artery is then dissected from its origin and the arterial continuity is maintained by its transposition into the right common carotid artery. This approach is minimally invasive, spares the need for sternotomy and also restores the circulation to the right upper extremity. Most patients report immediate resolution of their dysphagia and are able to tolerate a regular diet (7, 8).

In patients with severe symptoms who are poor surgical candidates, endoscopic dilation of the esophageal narrowing has been used to temporarily relieve symptoms of dysphagia. The goal of this procedure is palliative rather than curative (9). Since the compression is extrinsic in nature, the benefit of esophageal dilatation is very limited and short term. The use of self-expanding esophageal stents for treatment of malignant dysphagia as a palliative measure has been studied for several years; however, there are very limited data on stent placement in patients with dysphagia lusoria. Few studies have shown that stent placement in patients with malignant dysphagia (intrinsic or extrinsic compression) is a relatively safe, quick, reliable, and well-tolerated approach which provides immediate relief and palliation of dysphagia (10, 11). More studies are necessary to determine the success rate and long-term benefits of esophageal stent placement in patients with dysphagia lusoria.

## Conclusion

This case illustrates that congenital diseases can present later in life and are often missed unless appropriately suspected and pursued. Dysphagia lusoria is a rare, congenital anomaly that can be treated by various surgical or palliative techniques with high rates of success.
